# Extracellular Vesicles: Diagnostic and Therapeutic Applications in Cancer

**DOI:** 10.3390/biology13090716

**Published:** 2024-09-12

**Authors:** Maria Antonietta Di Bella, Simona Taverna

**Affiliations:** 1 Department of Biomedicine, Neurosciences and Advanced Diagnostics, University of Palermo, 90133 Palermo, Italy; 2 Institute of Translational Pharmacology (IFT), National Research Council (CNR), 90146 Palermo, Italy; simona.taverna@ift.cnr.it

**Keywords:** extracellular vesicles, cancer, biomarkers, diagnosis, therapeutic applications

## Abstract

**Simple Summary:**

Extracellular vesicles represent a heterogeneous family of lipid bilayer-enclosed particles naturally released by all cytotypes into the extracellular space. They can act as mediators of cell–cell communication. Recently, the applications of extracellular vesicles (EVs) have been of great interest in translational cancer research. The advances in next-generation omics technologies allow us to discover EVs’ selective cargo packaging and as EV roles in horizontal transport, non-invasive biomarkers, and as new therapeutic options. Herein, discussing recent studies, information on extracellular vesicle (EV) biogenesis, morphological characteristics, isolation, and current detection methods is summarized.

**Abstract:**

In recent years, knowledge of cell-released extracellular vesicle (EV) functions has undergone rapid growth. EVs are membrane vesicles loaded with proteins, nucleic acids, lipids, and bioactive molecules. Once released into the extracellular space, EVs are delivered to target cells that may go through modifications in physiological or pathological conditions. EVs are nano shuttles with a crucial role in promoting short- and long-distance cell–cell communication. Comprehension of the mechanism that regulates this process is a benefit for both medicine and basic science. Currently, EVs attract immense interest in precision and nanomedicine for their potential use in diagnosis, prognosis, and therapies. This review reports the latest advances in EV studies, focusing on the nature and features of EVs and on conventional and emerging methodologies used for their separation, characterization, and visualization. By searching an extended portion of the relevant literature, this work aims to give a summary of advances in nanomedical applications of EVs. Moreover, concerns that require further studies before translation to clinical applications are discussed.

## 1. Introduction

Cancer is one of the primary causes of illness and death in the world. Cancer cells are characterized by high proliferation rates; evasion of growth suppressors; the ability to self-renew; cancer stem cell features, inducing angiogenesis and metastasis; genome instability; and the capacity to switch between different metabolic pathways to acquire drug resistance [1].

To improve patient outcomes, one of the most challenging issues is to find innovative solutions for early detection and efficient treatment. In cancer therapeutics, the application of nanoparticles can represent new alternatives to chemotherapy or radiotherapy, overcoming the limits of these treatments [2,3,4], and precisely deliver therapies to cancer cells [5]. Currently, bioactive molecules are enclosed in nanocarriers to deliver drugs to a tumor site, targeting tumor cells in an efficient and selective manner [6]. Several organic and inorganic formulations have been produced, with encouraging results. However, these artificial nanocarriers have significant biases related to their in vivo toxicity [7]. To overcome these limitations, the possibility of using “natural vehicles” as nano shuttles is now being analyzed given their good quality. Among natural nanoparticles, extracellular vesicles (EVs) are heterogeneous nanosized vesicles released constitutively into the extracellular space in a way that has been conserved during evolution by almost all cytotypes in both prokaryotes and eukaryotes [8,9,10,11].

1. EVs have an aqueous core enclosed by a membranous lipid bilayer which contains several biomolecules that can be delivered to nearby or distant target cells. Since EVs play crucial roles as messengers over long and short distances, they can influence the microenvironment through the direct and protected shuttling of bioactive molecules, such as receptors and effector molecules, that can be protective or pathogenic [12,13,14].
2. EVs are involved in various physiological processes (apoptosis, differentiation, and proliferation), but they can also affect different pathophysiological conditions in the body, including inflammation, infection, immune response, and cancer [15,16,17,18].
3. Of natural origin, EVs exhibit low toxicity, biocompatibility, and high stability in the blood, due to their ability of immune system escape. Another EV advantage is their target potential [19].

**Figure 1:**
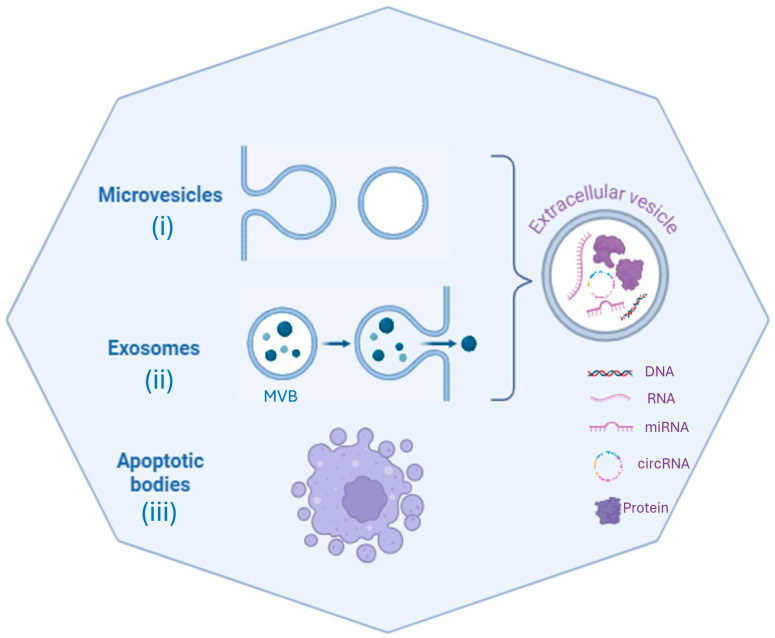
Biogenesis of EVs with biological molecules enclosed within a membrane: (i) microvesicles budding directly from the plasma membrane (microvesicles); (ii) exosomes released through fusion of multivesicular bodies (MVBs) with the plasma membrane; (iii) blebbing of larger vesicles from apoptotic cells (apoptotic bodies). The umbrella term of extracellular vesicles collects microvesicles and exosomes which contain several bioactive molecules: DNA, RNA, miRNA, circRNA, and protein. Image created in Biorender.com.

According to their size, composition, biogenesis prior to release, and function, EVs have been traditionally classified into three main types (Figure 1):

- Apoptotic bodies (1–5 µm) released by cell membrane blebbing during apoptosis.
- Microvesicles (150 nm–1 µm) shed directly by the plasma membrane with a mechanism of outward budding and fission.
- Exosomes (EXOs) (30–150 nm) originating from late endosomal trafficking [15,20,21,22].

Currently, EVs lack a uniform standard classification; the umbrella term, EV, is recommend for the various types of cell-derived vesicles, unless specific EV markers exclusively distinguish the origin of vesicles [23]. The International Society for Extracellular Vesicles (ISEV) first published the guidance recommendations for studies on EVs in 2014, then updated them in 2018, revising them in 2023 [24,25,26].

The position paper of the ISEV recommended using the term EVs for “*all particles released from the cell that are delimited by a lipid bilayer and cannot replicate*”.

To date, the scientific community is oriented to use the operational terms of small EVs to describe vesicles < 200 nm in diameter and large EVs for particles with diameter sizes > 200 nm, distinguishing them based on diameter size [25].

Small EVs (sEVs), owing to their attractive features, such as ultrasmall dimensions, spherical shape, and molecular components, have garnered great interest. Moreover, sEVs have the ability to cross physiological barriers, such as the blood–brain-barrier (BBB), and accumulate at pathological sites [27,28,29]. EVs are isolated, in physiological and pathological conditions, from blood and several body fluids, such as saliva, urine, cerebral spinal fluid, breast milk, ejaculate, amniotic fluid, and other malignant effusions [16,30,31,32,33,34].

EVs are considered a very promising source of circulating biomarkers; they could find application in the prevention, diagnosis, prognosis, and treatment of cancer [35]. Recently, EVs have shown key roles in the intricate field of cancer research. A growing body of research is concerned with understanding the pleiotropic roles of EVs in tumorigenesis and cancer progression. Conventional cancer biomarkers have low sensitivity and specificity, and their application in clinical practice is still limited [36].

This review, focusing on recent publications, reports how EVs can be considered a new class of biomarkers for cancer detection and monitoring. The review reports the new strategies for EV isolation and detection procedures, analyzing the advantages and limitations of these innovative technologies. Furthermore, we discuss the future directions of EVs for clinical applications. The source for the retrieval of the literature contained in this work was the PubMed database.

## 2. Small EVs’ Characteristics and Biogenesis

The following section is focused on the small vesicles called exosomes (EXOs) and summarizes their characteristics.

**Figure 2:**
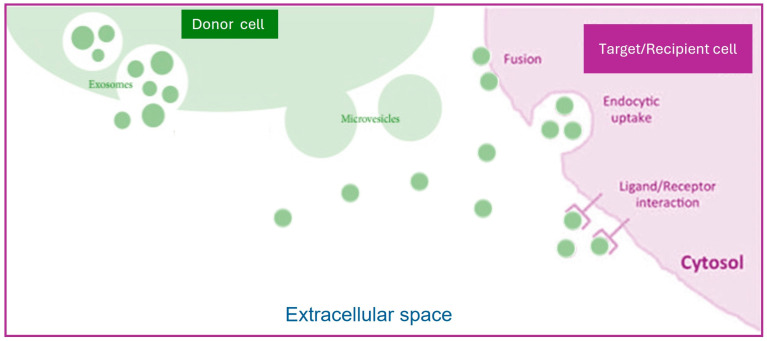
Schematic representation of EV internalization. Exosomes and microvesicles released into the extracellular milieu from donor cells can be uptaken by target cells in three diverse ways: direct fusion with the plasma membrane, endocytosis, and protein–receptor interaction.

- *History*: EXOs were isolated for the first time in 1983 from a sheep reticulocyte culture supernatant. For many years, EXOs were considered as waste obtained from plasma membrane shedding. After several decades, this image of a bin to remove garbage from cells changed to that of a shuttle for bioactive particles. Later, the term “exosome” was coined to distinguish them from other types of EVs [20,37,38,39]. After these initial reports, the EXO research field has garnered much interest and has been enriched considerably, as documented by the exponential increase in publications that has occurred since 2010, according to PubMed.
- *Composition*: Complex biological molecules of different kinds are found in EXOs; these bioactive molecules are selectively packaged on an EXO’s surface or inside the lumen. Omics approaches (proteomic, lipidomic, metabolomic, and deep sequencing technologies) allowed the discovery of the proteins, lipids, metabolites, and nucleic acids contained in EXOs.
  Based on specialized databases, such as EXOCARTA (http://www.exocarta.org), exoRBase (http://www.exorbase.org), EVpedia (https://evpedia.info), Vesiclepedia (http://www.microvesicles.org), EV-TRACK (https://evtrack.org), and ExoBCD (https://exobcd.liumwei.org), it is possible to collect information about EV cargos [40,41,42,43,44]. EXOCARTA (accessed on 1 June 2024) reports that EXOs contain 9769 proteins, 3408 mRNAs, 2838 microRNAs (miRNAs), and 1116 lipids [45,46]. Protein cargos are varied among EVs due to the specific features of the cell types, culture conditions, and isolation procedures. Some proteins are present mostly in EXOs, being specific to biogenesis and protein sorting; thus, they can be used as markers for EXO characterization [47]. Proteins include both membrane and cytosolic components, such as surface receptors, adhesion proteins, integrins, cytoskeletal proteins, membrane transport proteins, and fusion-related proteins. One of the reasons for EXO heterogeneity is the presence of specific proteins that mirror the status of the parental cell. For instance, EXOs released by T-lymphocytes have enzymes and perforin on their surface. Antigen-presenting cells (B-lymphocytes and dendritic cells), APC-derived EXOs, contain major histocompatibility antigen complexes (MHC, MHC-I, and MHC-II) [48]. The tetraspanin proteins (CD9, CD63, CD81, CD82, and CD86) are localized on EXO membranes and represent hallmarks of them. Alix, flotillin, and TSG101 are involved in their biogenesis [49]. EXO intravesicular proteins are heat shock proteins (Hsp20, Hsp27, Hsp60, Hsp70, and Hsp90), cytoskeletal proteins that are the most conserved molecules; transcription factors (Wnt, Notch, and hedgehog), transport and fusion proteins (GTPase), cell-surface peptidases (CD13 and CD26), and signaling receptors like EGFR are abundant in EXOs [17,50]. Nucleic acid cargos are very abundant in EXOs and are variably expressed in different disorders. They contain both DNA molecules, such as double-stranded (dsDNA), single-stranded (ssDNA), and mitochondrial DNA (mtDNA), and RNA [51,52]. EXOs are enriched with several different species of RNA, such as messenger RNA (mRNA), transfer RNA (tRNA), 18S and 28S ribosomal RNAs (rRNAs), microRNA (miRNA), Y-RNA, long non-coding RNA (lncRNA), circular RNA (circRNA), small nuclear RNA (snRNA), small nucleolar RNAs (snoRNAs), P-element-induced wimpy testis (PIWI)-interacting RNAs (piRNAs), and viral RNA [53]. Hundreds of lipid species are found in EXO membranes; sphingomielyn, desaturated lipids, and cholesterol, abundantly present, are responsible for the stability of these vesicles [54,55]. Ceramide phosphates seem to be involved in the anti-inflammatory function of EXOs [56,57].
- *Formation:* EXOs originate from the endo-lysosomal compartment. As a first step, plasma membrane invaginations generate early endosomes that mature to late endosomes, producing multivesicular bodies (MVBs). The latter contain intraluminal vesicles (ILVs) formed after invagination of the late endosomal membrane and enclosing cargo inside [58]. Subsequently, MVBs may bind to the plasma membrane, and EXOs are released into the extracellular space. To develop ILVs, ESCRTs (endosomal sorting complexes required for transport) are essential. Protein ESCRTs 0-III are involved in promoting MVB formation, vesicle budding, and sorting of protein cargo [59]. An alternative ESCRT-independent mechanism including roles for tetraspanins, and lipid rafts is also required.
- *Release:* To release EXOs into the extracellular space, MVBs fuse with the plasma membrane. The process is coordinated by RAB proteins and their effectors, or Snap receptors. Without these interactions, MVBs will be degraded into lysosomes [59].
- *Uptake:* EXOs released into the extracellular space deliver their cargo to the recipient cells or can be destroyed. The target cells may be in proximity to or distant from the parental cells. EXOs can travel over large distances via blood or lymphatic circulation; their lipidic membrane is a protection barrier for the cargo, and the signals travel protected from degradation, avoiding phagocytosis. Molecules such as nucleic acids that could be degraded in extracellular spaces are protected from enzymatic degradation (by RNAse, for example) [13]. The cargos enter through three major mechanisms: endocytosis uptake, direct fusion to target cell membranes, and receptor–ligand binding (Figure 2). The recipient cells can internalize EXOs as whole vesicles that surf on filopodia to reach endocytic hot spots; then, they pass through endosomes and are finally targeted to the endoplasmic reticulum [60,61,62,63].
- *Function:* EXO cargos, released inside target cells, can act in appropriate cellular compartments, performing various functions that, depending on the cellular origin, regulate a plethora of activities. EXOs may modulate gene expression, metabolism, and responses to microbial infection and enhance disease progression [23]. It has been demonstrated that EXOs may transfer nucleic acids, such as mRNA and miRNA, between cells and facilitate their translation in target cells [64]. EXOs generated from dendritic cells can alter the immune cell response of dendritic cells through the transfer of MHC classes I and II [65]. The amount of released EXOs depends on the parental cell’s physiological and/or pathological states. Cell stress and the activation of several pathways can regulate EXO production. According to several reports, cancer cells release more EXOs than normal cells. Specific tumor antigens and miRNAs found in EXOs can promote cancer progression by activating oncogenic and anti-apoptotic pathways, such as invasion, angiogenesis, and metastasis [66]. EXOs also promote tumor immune escape with T-cell apoptosis induction. They have important roles in epithelial–mesenchymal and mesenchymal–epithelial transition in different malignancies. Thus, it is crucial to know the biological conditions of parental cells to guarantee the safety of these vesicles in clinical applications [18,67,68,69,70] (Figure 2).

## 3. EV Isolation and Storage Methods in Cancer Diagnostics

Currently, EVs can be isolated using different methods, depending on their properties, such as size, shape, density, and surface molecules. Conventional and new technologies are sometimes not capable of characterizing EVs and providing complete qualitative and quantitative information. These methods need to be selected based on the need and further downstream applications. When animal and human EVs are isolated for further analysis, a complete body fluid sample that contains cells, microvesicles, platelets, DNA, RNA, and proteins, must be isolated and used for detection. The isolation of tumor-derived EVs and their separation from normal EVs continues to be particularly difficult. The new detection and analytical techniques are often employed for comprehensive characterization. Since no available purification method can strictly separate EVs based on their size, a step of characterization is needed. MISEV2023 highlighted the importance of proper characterization of EV fractions and recommended the use of combined methods for EV extraction and new techniques for EV purification in order to validate and replicate experimental results [25].

### 3.1. Conventional EV Isolation Methods

Different methods and procedures have been established to isolate EVs from in vitro cell culture supernatants, biological fluids, and non-human sources such as plants and other species. It is crucial to evaluate an EV isolation method according to the sample type, volume, and research question. Depending on the purposes and applications, it is particularly important to select the best EV producer cells, and it is essential to know the features of the different isolation approaches that allow large-scale biomanufacturing and guarantee the efficacy of the strategy. Indeed, certain procedures may compromise EV structure and integrity or cause aggregate formation and cargo impurity [71].

**Figure 3:**
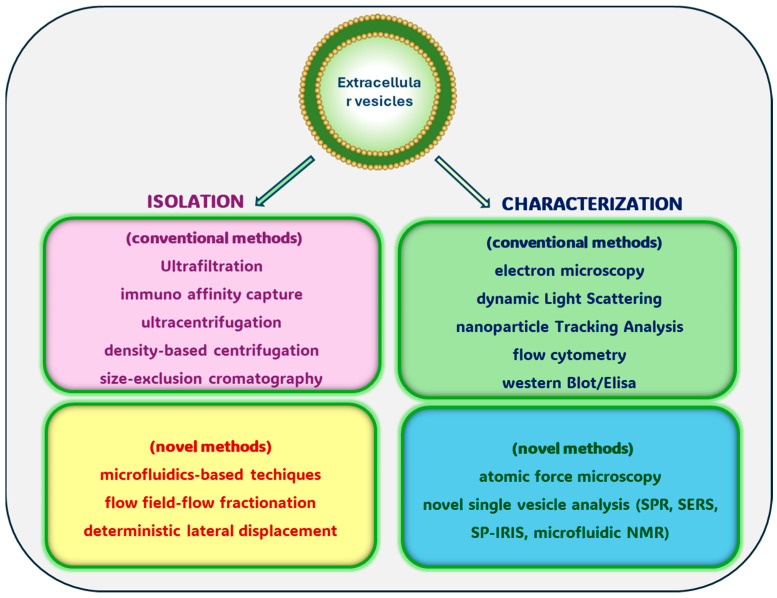
Conventional and novel techniques of isolation and characterization of EV samples.

Among the several isolation procedures, the most traditional and common are the following:

- *Ultrafiltration (UF):* This technique is a passive isolation method that utilizes membranes such as polyether sulfone (PES) with predetermined, extremely small pores (100 nm diameter) to isolate vesicles based on their size and molecular weight [72]. The method involves the use of fluid pressure to drive the migration of a sample through a polymeric filter. EVs are isolated selectively from samples with simultaneous retention of larger particles. UF is a simple and fast method of low cost. However, the applied pressure in filtering the material can damage EVs via shear stress. The result is loss of vesicles; membrane pore blockage, due to the accumulation of particles with the interaction between vesicles and membranes, is also a noticeable problem, as it can reduce the lifetime of membranes and leads to lower EV purity and low efficiency. This reduces UF efficiency, and although UF might be sufficient for good EV isolation, better selectivity and purity can be achieved with a combination of UF and other techniques [73,74].
- *Immunoaffinity capture (IC):* This technique is an isolation technique based on the specific recognition and binding between antibodies or affinity ligands (such as lectins and heparin) and EV marker components (antigens) that are ubiquitously exposed on vesicle surfaces. The approach is simple, as the antibodies or ligands are immobilized on solid substrates such as magnetic beads. A sample is incubated with these beads, and they are captured through specific antigen–antibody binding or ligand–receptor interactions. The magnetic beads are separated from the other molecules contained in the sample using a magnetic field. A washing step with buffers reduces the non-specific interactions, and after elution the vesicles can be used for further analysis [75]. EV surface markers, such as fusion proteins (flotillin and annexin) and transmembrane proteins (CD63, CD81, and CD82), can be recognized by antibodies that are applied to separate vesicles from various sources. Several commercial kits have been developed over the past decade for selectively isolating EV subpopulations. Using specific biomarkers such as EpCAM (epithelial cell adhesion molecule) overexpressed on tumor-derived EVs, researchers were able to isolate EVs from clinical samples and evaluate the presence of marker-related tumors [76]. The method based on magnetic beads can achieve strong specificity and good purity and yields; it can be used with small volumes of a sample; and it is useful for the separation of cell-type-specific EVs [77]. For instance, the isolation of CD9^+^ EVs can lead to the exclusion of CD9 EVs, which can be used for diagnostic purposes. A disadvantage associated with this methodology is the selection by the users of a subset of marker-specific vesicles that may not reflect all EVs. A limit on its large-scale use is the availability of specific and good antibodies and the high reagent cost. Indeed, immunoaffinity capture is one of the most expensive methods of EV isolation from a large sample volume, as it requires high amounts of antibody-conjugated beads. Therefore, it is only appropriate for small sample sizes, which presents a barrier to any potential therapeutic use. In the past few years, several isolation platforms based on aptamers have been developed [78,79]. Aptamers are short DNA or RNA oligonucleotides that can recognize and bind targets in a manner like antibodies. In comparison with antibodies, they can be produced by chemical synthesis in vitro, present low or no immunogenicity, and be low cost. Therefore, they can be used to detect EVs from cancer cells, as they bind with high affinity and specificity. Aptamers can bind EVs with high specificity; however, they have not been widely used for EV isolation, being used mainly for analysis and detection [80].
- *Ultracentrifugation (UC):* Ultracentrifugation (100,000× *g* or greater) is currently used in about 80% of EV isolation methods. It is based on the principle of sedimentation and mainly depends on vesicle density, size/weight, and shape, as denser and/or larger particles tend to fall to the bottom first [81,82]. UC involves two steps with increasing centrifugal power after pelleting down cells: first a pre-cleaning and filtering of samples centrifuged at low and intermediate speed centrifugations (500–2000× *g*) to remove dead cells and large debris; then, the pellet is resuspended in a suitable medium and centrifuged at 10,000–16,000× *g* to pellet apoptotic bodies [83]. After removing these large species, via ultracentrifugation at 20,000× *g*, a pellet enriched in MVs, a subset of large-sized extracellular vesicles, can be obtained. The supernatant is then filtered through a 0.45–0.22 μm filter (Millipore) to reduce potential MV contamination and centrifuged at high speed (40,000–100,000× *g*) for several hours. EVs are separated from different components of the sample, pelleted at the bottom of the tube, and collected after removing the supernatant. The centrifugation is operated at 4 °C. UC presents several advantages: it requires little sample pre-treatment, and it is suitable for processing large samples (depending on the rotor utilized), such as cell culture media and easily accessible biofluids, and isolating large amounts of EVs [82]. Furthermore, UC has the characteristic of low contamination risk and is also inexpensive, apart from requiring access to expensive equipment (purchase of an ultracentrifuge). At the same time, however, there are several limitations because UC isolation is a time-consuming method (>4 h) and requires extra care to prevent damage from the centrifugal force. UC may result in partial vesicle aggregation and degradation and can also lead to the loss of some of the EVs’ original biomolecular contents [81,84]. In addition, the resulting supernatants obtained by ultracentrifugation at 100,000× *g* may contain non-vesicular extracellular nanoparticles, such as exomeres and supermeres, which were recently discovered and seem to contain and transfer functional cargos [85]. As regards human plasma, because of the considerable overlap in terms of both particle size and density between EVs and lipoprotein particles, such as low-density lipoproteins (LDLs), very-low-density lipoproteins (VLDLs), intermediate-density lipoproteins (IDLs), and chylomicrons, an unintentional coisolation of these two different entities may occur. Hence, ultracentrifugation is not widely recommended to purify EVs for therapeutic purposes, as it may coisolate protein aggregates [86].
- *Density-gradient centrifugation (DGC):* To eliminate impurities, such as proteins, lipoproteins, and RNA, from biological fluid, high-speed centrifugation and density gradients are often combined. These procedures involve the use of centrifuge tubes filled with an inert medium such as sucrose or iodixanol (Opti Prep) that decreases in thickness from bottom to top [87,88]. DGC utilizes differences in densities between particles and media. The process starts by filtering or centrifuging a sample to remove debris and large particles. The sample is loaded on top of the density gradient and centrifuged at high speed for several hours [89,90]. EVs are separated according to their density, size, and mass. Each sample component migrates according to its density in the tube’s pre-loaded density gradient, causing separation to occur [91]. EVs are localized in a density range of 1.1 to 1.2 g/mL in sucrose density gradients. The EV aliquot is collected from the required density range; the sample is ultracentrifuged at 100,000× *g*, for a few minutes, to yield pure EV pellets. Depending on the acceleration, the type of rotor in which the samples are placed, the viscosity of the solution in question, and the time needed to obtain the pellets, preservation of vesicular structures with high purity is obtained. Compared to differential ultracentrifugation, density-gradient centrifugation gives a cleaner EV population. However, it has several limitations: DGC requires specialized equipment and can be expensive and time intensive. Moreover, some contaminants can persist because EVs cannot be isolated from vesicles with the same density but different sizes [92]. Because of the long time required, DGC is not applicable for use in clinical settings or efficient enough for biomarker discovery, but it is an important technique in EV research.
- *Polymer-based precipitation:* The coprecipitation method is a simple, quick, and efficient way to isolate EVs. It uses hydrophilic polymers where the sample is incubated. Polymers at low-speed centrifugation sequester the water molecules so that the solubility of the soluble components decreases, and they are then forced to phase separate. EVs or biological material are excluded from the solvent regions and are concentrated until, their solubility being exceeded, they precipitate [93]. Currently, among hydrophilic polymers, polyethylene glycol (PEG), lectin, dextran, or salt solutions are used. After washing, and using a neutralizing agent, EVs are separated by low-speed centrifugation (1500× *g*) [94]. This technique is used to separate EVs from biological fluids such as plasma, urine, and cerebrospinal fluid. PEG is used to process many samples simultaneously; it is easy to use, fast, does not require special equipment, is relatively low-cost, and does not cause deformation or damage to EVs. Commercial kits, such as Total Exosome Isolation, ExoSpin, and ExoQuick, are based on polymer precipitation and are available for scaling up. However, the low specificity and coprecipitation of different components like protein pollutants, polymeric materials, and lipoproteins may occur, which can lead to incorrect quantification of EVs, that is generally dependent on total proteins, which limits further analysis of EVs via omics-based assays. It is an attractive tool for rapid isolation, can be applied for preliminary analysis, and is efficient in clinical research settings, but it is not considered for functional analysis [95].
- *Size-exclusion chromatography (SEC):* This technique, also referred to as gel filtration, has exploded in popularity in recent years. Using passive gravity flow, it can isolate solutes based on molecular size, shape, and density. The method includes the passage of an aqueous solution down a column filled with a porous unreactive stationary phase and a specific pre-size distribution to differentially enable elution of the material. When the sample, the initial biofluid, enters the gel, with the flow of the mobile phase, small particles or molecules with small hydrodynamic radii diffuse into the pores and are trapped in them for a long time, so they pass slowly through the column. Conversely, particles with large hydrodynamic radii are unable to access the pores, so they are eluted earlier from the column. Hence, the passage of proteins and other small contaminating particles is delayed, while larger particles or vesicles like EVs exit the column and are eluted earlier in the void volume [96]. The separated EVs are collected in fractions to be used for experiments. The porous stationary phase contained in the column can be a cross-linked dextran polymer (Sephadex), polyacrylamide (Sephacril), agarose beads (Sepharose), or allyldextrane (Sephacryl). In the last decade, many commercially available columns and SEC kits have been designed to simplify EV isolation: iZON Science produced the qEV Exosome isolation kit; the PURE-EVs kit (Hansa Biomed) has also been created. These systems allow rapid isolation with high precision in half an hour; the SEC methodology is relatively easy and fast [97]. SEC is useful to isolate EVs from a large variety of sample matrices from both prokaryotes and eukaryotes. This technique allows the isolation of high-purity EVs, preventing protein contamination, and the maintenance of the integrity of EV structures and biological activities, as this procedure relies on gravity rather than sheer force; it is suitable for use with small samples, efficient in terms of time (<0.5 h) and effort, and there is no or minimal sample loss. Therefore, SEC has potential for therapeutic applications and functional investigation [98]. The SEC approach requires dedicated equipment and has several limitations: (a) due to the possibility of contaminants gaining access to the chromatography columns, it is important to ensure aseptic working conditions, especially if the isolated EVs are destined for therapeutic use (to eliminate soluble contaminants, filtration-based techniques are used prior to injection into the column) [99]; (b) for the starting material, a medium sample volume is required; (c) the number of samples that can be processed simultaneously is another limitation associated with SEC; (d) low yield, due to the inability to separate EVs from other vesicles of the same size or protein aggregates or lipoproteins (including chylomicrons, VLDL, VLDL remnants (such as IDL) and LDL) in the same size range as EVs [100]. The identification of EV subtypes is important, and the combination of SEC and immunocapture methods is recommended. Research efforts have been made to overcome these challenges and enhance SEC efficacy and speed [82,96]. For instance, using a combination of UF/UC or PEG-based precipitation and SEC to remove pollutants makes it a powerful isolation technique for EV research, especially if subsequent downstream therapeutic and biomarker discovery applications are planned. SEC-coupled techniques generate a high yield of EVs that can be used for protein and RNA diagnostics, as well as potential drug or drug delivery systems. However, this combination target is not suitable for scaled-up production [97,101] (Figure 3).

### 3.2. Advanced EV Isolation Techniques

Other advanced and efficient separation procedures that allow the isolation of EVs based on their physical and biochemical features simultaneously are being developed. There are isolation methods that need small starting volumes from cell culture supernatants or serum/plasma (10–100 µL); other fast and efficient methods can be performed on larger starting volumes, reducing reagent consumption.

- *Microfluidic-based platforms (MF):* Microfluidic-based platforms are new isolation methods that are emerging due to their small size, automation, efficient and rapid enrichment, and isolation of particles of very similar shapes and sizes. They require only small volumes, but the devices are highly complex and expensive, although they are less expensive than those used for immunoaffinity capture. Microfluidics is a modern technology with promising prospects and has great potential in clinical applications, but it is not yet considered a standardized method of EV isolation. Different isolation principles have been designed: size-based, immune-affinity-based, and dynamic categories that make use of nanomaterials or polymethylmethacrylate (PMMA) [102].

*Size-based EV separation devices:* Passive isolation methods allow vesicle separation according to their size, via filtration. Driving a sample inside a microchannel-integrated membrane using nanoporous in situ filters predesigned for a specific size, ultrathin nanoporous membrane chips, or nanoarrays and nanowires, EVs can be trapped when fluid flows through them. Diffusion coefficient and sedimentation velocities are the parameters that function to separate big particles from smaller ones. EVs can diffuse faster, while molecules larger than the pores of the nanoporous filtering membrane cannot pass through, remaining at the bottom of the inlet chamber [103,104]. The ExoTIC chip was designed by Liu et al. to isolate vesicles from different biological samples [105]. This procedure can be easily used, as it does not require user training, and it is appropriate for isolating EVs from small samples, such as saliva, plasma, tears, and culture media, making it suitable for clinical tests [106]. Major drawbacks of this technique are blockages and the requirement for frequent filtering and channel clogging when volumes of samples are not small.

*Immunity-affinity based microfluidic devices:* Vesicle separation relies on specific binding in an analogous manner to the general methods of immunity capture described before. A sample flows through microfluidic chips of polydimethylsilaxane (PDMS) that are functionalized with antibodies. One of these commercial affinity systems is the Exochip^TM^. It consists of microfluidic multiple circular capture chambers interconnected by narrow channels and coated with an antibody against the EV surface CD63 marker, a commonly expressed antigen. The vesicles are captured by the highly selective and specific antigen–antibody binding and are efficiently retained [107]. This method is very fast, easy to use, and efficient. Zhao et al. developed a microfluidic device (the ExoSearch chip) for continuous exosome isolation and detection from human plasma using magnetic beads conjugated with three antibodies against common exosomal markers (CD9, CD81, and CD63) for immunocapture and fluorescence-labeled tumor markers (CA-125, EpCAM, and CD24) for probing [108]. Sample volumes as low as 10 µL can be utilized for isolation. Moreover, the major advantage of this approach is the possibility of isolating EVs from different sources, allowing the discrimination of tumor patients from healthy controls by the quantitative detection of EVs, which makes it attractive and suitable for clinical applications and diagnostic purposes. However, several limitations still persist: the high cost of good-quality ligands; the requirement of specialized equipment; the need for highly represented target proteins on the surface vesicles; and the need to know the molecular composition of the target vesicles [109]. Non-specific binding may be reduced with the use of monoclonal antibodies. The EVs bound to the antibodies should eventually be removed by dissolving them in a solution, which can contaminate the collected vesicles. Efforts to solve those problems have been made by researchers [110].

*Microacoustic fluidic devices:* The active isolation method enables the separation of EVs directly from undiluted small samples in a contact-free mode. A sample is placed in a chamber that is subjected to a continuous flow of ultrasound standing waves. Without contact, based on the size, density, and composition of the membrane, the EVs react to the acoustic radiation forces and move toward the chamber, forming clusters; these are then washed and released upon deactivation of the ultrasound. EVs separated by this device maintain their structures, characteristics, and functions. This device requires short processing times and little operator intervention. The original volume of the sample may be very small [111]. EV purities of about 98% can be reached; thus, it appears to be suitable for clinical applications.

*Dielectrophoretic microfluidics (DEP):* A sample, without pre-treatment or dilution, is subject to an alternating current, and EVs with a degree of polarization move toward the electric field and can be separated and concentrated by their size and dielectric properties. DEP allows the obtainment of even deeper information on the properties of both charged and non-charged EVs. It has been successfully utilized to isolate EVs from blood, serum, and plasma in less than 30 min [112]. In 2021, Zhao et al. developed a device (the ExoDEP chip) for exosome isolation and detection [113]. Another active isolation approach is represented by the electrophoretic and electromagnetic methods. Vesicles are separated from a sample based on their electrophoretic mobilities using an alternating current electrokinetic (ACE) microarray chip [114]. ACE chips are useful for the rapid isolation and detection of EVs; however, other entities such as protein aggregates and cell debris can also separate along with EVs under the applied AC electrical field. In addition, the contact with the electrodes and the high operational voltage may modify the properties of the sample. Therefore, isolation and accurate detection of pure EVs by this method are challenging.

*Deterministic lateral displacement (DLD):* This is a microfluidic technique that separates EVs based on their trajectories in a pillar array with a regular arrangement [115,116]. Particles smaller than the set DLD critical diameter will follow a zigzag direction, while larger particles travel in a bumping or displacement mode, resulting in separation based on their size differences. DLD pillar arrays with 235 nm nanopillar gaps are used to separate EVs with sizes in the range of 20–110 nm [117]. Due to its low cost, simplicity, robustness, particle separation, and detection capabilities, microfluidic DLD seems that it could have a potential impact on point-of-care diagnostics in the future.

- *Flow field-flow fractionation (FFF):* This is an emerging passive size-based fractionation technique for EV separation based on the application of hydrodynamic forces. Asymmetrical flow field-flow fractionation (AsFlFFF or AF4) is the most popular FFF subtechnique used to fractionalize EVs from cell-line culture media and body fluids with high reproducibility and purity [118], although it is more expensive than the others.
- *Ion-exchange techniques:* These allow the isolation of EVs, for instance, through chromatography- and metal-affinity-based systems that exploit the interactions between negatively charged EV membrane components whose charges have been determined by zeta potentials and an anion exchanger with positively charged functional groups or cations [119].

Figure 3 summarizes the EV isolation and characterization methods.

The storage conditions and preservation of isolated EVs for therapeutic applications need to be fully elucidated, as they can affect the amount, size, cargo, functions, performance, and quality of the final product. When cryoprotectants (such as human albumin, trehalose, and dimethyl sulfoxide) are added, the stability of EVs can be improved. Several data suggest that the stability of EVs of different origins may also be different; storage at −80 °C in phosphate saline buffer is the most used system to preserve EVs [120]. EV losses can reach 51% ± 3% when cell-culture-derived EVs are stored for 48 h at +4 °C in polypropylene tubes [121].

## 4. EV Characterization and Detection Techniques

Several procedures have been developed to characterize isolated EVs; the guidelines for EV characterization updated in MISEV2023 [25] are as follows:

(a) Quantifications in terms of the initial volume, source (such as a biofluidic sample), or mass of tissue abundance.
(b) Analysis of protein composition and lipid-bound or transmembrane-bound proteins.
(c) Application of two distinct complimentary methods, such as the use of single-particle analysis instrumentation and electron microscopy.
(d) Evaluation of the topology of EV-related components.

### 4.1. Commonly Utilized EV Characterization Methods

The conventional methods enable determination of the shape, amount, size, and surface protein composition; however, they alone are not sufficient to provide complete qualitative and quantitative information. Traditional approaches involve the use of Nanoparticle tracking analysis (NTA), Dynamic light scattering (DLS), Resistive pulse sensing, Electron microscopy (EM), and Atomic force microscopy (AFM), which are commonly employed methods for size and concentration measurement. Biochemical characterization of EVs is usually performed using immunoblotting, proteomic analyses, and flow cytometry. Some of these approaches need skilled operators and sometimes are laborious and time-consuming. The most used of these current conventional detection methods and their characteristics, merits, and pitfalls are summarized in Table 1, and readers may refer to references [122,123,124,125,126,127] for more details.

### 4.2. Novel Single-Vesicle Analysis Methods

This section highlights some novel single-vesicle analysis methods involving new microfluidic techniques used to isolate, detect, and quantify EVs in mobile systems. They provide efficient detection, high precision, and low reagent consumption. To understand the role of EVs, it is important to analyze their chemical composition. Traditionally, labelling techniques such as fluorescent or radioactive tags, which can specifically target molecules within EVs, have been used. However, these methods can be time-consuming and expensive and can alter the natural composition of EVs. By contrast, label-free techniques do not require any EV modification and can provide information on their natural chemical composition [128].

- *Surface plasmon resonance (SPR) detection:* SPR occurs when light incident at a certain angle through a glass prism strikes a metal sheet at the interface of two substances with different refractive indices. The resonance condition is strongly dependent on the refractive index of the media. This real-time analysis technique can thus detect molecular interaction on the surface of a gold layer by monitoring the changes in its refractive index [129]. Rapid real-time labelling detection can be achieved using plasmon resonance systems, which do not rely on secondary antibody labelling [130]. Plasmas can squeeze and enhance electromagnetic fields on the subwavelength scale, which is widely used in optoelectronics and quantum information, as well as biomedicine [131]. SPR may be used to detect and measure particle concentrations of EVs within 200 nm of the metal film. This technique provides various advantages, such as low cost, rapid detection, low sample consumption, specificity, and sensitivity. However, some limitations need to be overcome to make this method suitable for clinical application.
- *RAM surface-enhanced Raman spectroscopy (SERS)* and *Tip-enhanced Raman spectroscopy (TERS):* These are powerful techniques to enhance the Raman scattering signals of the molecules adsorbed on or proximal to metal surfaces, semiconductors, and 2D nanomaterials. Particularly useful for studying complex biological systems, such as cells and extracellular vesicles, SERS and TERS are useful where traditional Raman spectroscopy may not provide sufficient sensitivity for identifying EV biochemical compositions and their membranes, allowing the detection of small molecules, lipids, and proteins. For instance, EVs contain helical fragments of proteins on their outer membranes. Upon EV interaction with gold nanoparticles or a gold-tip apex, they result in signal amplification, offering molecular specificity, high sensitivity, high spatial resolution, non-destructiveness, and versatility [132].
- *Single-particle interferometric reflectance imaging sensor (SP-IRIS) (Exo-View):* This is a digital optical technology which enhances nanoparticle scatter signals by the application of extra layered substrates (silicon substrates). Based on this method, the Exo-View platform has been used to characterize different ratios of surface markers like tetraspanins, differentiating EV populations produced by cancer cells. It can detect EVs as small as 40 nm, and it can be used for the analysis of proteins of purified individual EVs [133].

To sum up, multiple techniques have been recognized to isolate and characterize EVs; however, scalability, validation, sample pretreatments, and standardization are still considered bottlenecks for these devices, which could largely be applied in cancer diagnosis [118,134].

## 5. The Role of EVs in Cancer Diagnosis: EVs as Biomarkers

To date, cancer is the second leading cause of death worldwide. The majority of solid tumors are usually diagnosed by tissue biopsy. The information gained from a traditional biopsy may not reflect tumor heterogeneity, and tissue biopsy cannot be performed routinely to monitor prognosis and treatments. Moreover, conventional tumor markers are often limited and inaccurate for clinical use. Thus, there is an urgent need for new and non-invasive approaches that can completely represent tumor features and allow early detection and accurate monitoring of prognosis and treatments [135].

In the following section, recent findings on non-invasive diagnostic tests are highlighted. Liquid biopsy (LB) is considered a highly promising and non-invasive diagnostic tool, facilitating early diagnosis, dynamic monitoring of prognosis, and treatment response. Moreover, LB can detect emerging drug-resistance mutations, guiding the selection of different therapeutic options. Cancer mutational profiles detected by LB analysis can provide a whole overview of cancer heterogeneity compared with conventional tissue samples. LB enables the identification of specific genetic variants, guiding personalized medicine selection, improving therapeutic efficacy, and minimizing side effects [136].

LB garnered special interest in the field of precision oncology, and its application is included in standard clinical management algorithms for some cancers [137]. LB samples can be obtained from body fluids, using simple blood draws, urine, cerebrospinal fluid collection, or sampling of malignant effusions. This feature is very important when tissue samples are not feasible to access, and LB repeated testing can be easily carried out without exposing patients to the hazards of tissue biopsy. LB samples contain an assortment of analytes useful as biomarkers, such as circulating tumor DNA (ctDNA), cell-free DNA (cfDNA), circulating tumor cells (CTCs), tumor-educated platelets, proteins, miRNAs, metabolites, and EVs. Currently, the three most widely studied LB-based biomarkers are CTCs, ctDNA, and EVs [138].

EVs can represent a new paradigm in cancer diagnosis. They can overcome CTC and ctDNA limitations. EVs are abundant in the circulation (about 10^10^ EV/mL plasma), and the lipid membrane protects their cargos (proteins, nucleic acids, lipids, and metabolites) from degradation. The lipidic bilayer contributes to the high bioavailability, low immunogenicity, and toxicity of EVs. EV cargos, such as protein, lipid, DNA, RNA, ncRNA, and other markers, have great potential as biomarkers. Recently, it was reported that non-coding-RNAs (ncRNAs), such as miRNAs and circRNAs, found in tumor-derived EVs deliver critical information for cancer detection [139].

EV-ncRNA amounts and compositions differ between diseased and healthy individuals. Aberrant ncRNA levels have been described in many malignancies; since tumor-derived EVs reflect the miRNA expression of originating tumor cells, these miRNAs have been considered as biomarkers in the plasma EVs of tumor patients that are useful for improving cancer diagnosis.

Fluctuations in EV-miRNA levels can be detected before patients develop evident symptoms of cancer [140]. Several studies describe the function of EV-miRNAs and their ability to serve as biomarkers for many cancer types, as reported in this section. EVs isolated from plasma of non-small cell lung cancer (NSCLC) patients and controls showed that ten miRNAs were significantly differentially expressed: Hsa-let-7 days, hsa-miR-223, hsa-miR-383, hsa-miR-572, hsa-miR-20b, hsa-miR-30e-3p, hsa-miR-301, hsa-miR-192, hsa-let-7f, and hsa-miR-345. Five of these (let-7f, miR-20b, miR-30e-3p, miR-223, and miR301) were randomly chosen for further clinical valuation on a new set of NSCLC patients and controls [141]. Only let-7f, miR-20b, and miR-30e-3p were significantly reduced in NSCLC patients’ plasma EVs. Increased levels of let-7f and miR-30e-3p differentiated patients with resection (stages I, II, and IIIA) from those without (stages IIIB and IV) [142]. A long-term follow-up indicated that high levels of miR-30e-3p and let-7f in plasma EVs were correlated with a higher rate of disease-free survival and overall survival, respectively.

Moreover, miR-223 is increased about 200-fold in EVs isolated from the plasma of NSCLC patients with respect to healthy controls. High levels of miR-30b, -30c, -103, -195, -221, and -222 were packaged in EVs, according to studies that describe the prognostic value of miR-103, -203, -30b-c, -221, and -222 as biomarkers of lung cancer [67,143].

EV-miRNAs are also deregulated in the blood of breast cancer patients compared to healthy individuals. For instance, miR-1246 and miR-21 were upregulated in EVs collected from the plasma of breast cancer patients compared to those of healthy subjects. Four miRNAs (miR-9, miR-16, miR-21, and miR-429) were found to be upregulated in the EVs of early-stage breast cancer patients compared to healthy donors [144]. Another study reported that 11 EV-miRNAs (miR-338-3p, miR-340-5p, miR-124-3p, miR-29b-3p, miR-20b-5p, miR-17-5p, miR-130a-3p, miR-18a-5p, miR-195-5p, miR-486-5p, and miR-93-5p) were also related to breast cancer recurrence without reflecting the expression in primary tumor tissues [145].

In patients with triple-negative breast cancer (TNBC) undergoing neoadjuvant chemotherapy, a signature of four EV-miRNAs (miR-4448, miR-2392, miR-2467-3p, and miR-4800-3p) was able to discriminate between patients with pathological complete response (pCR) and non-pCR [146]. Moreover, four miRNAs evaluated before the treatment (miR-30b-5p, miR-328-3p, miR-423-5p, and miR-127-3p) and four after (miR-141-3p, miR-34a-5p, miR-183-5p, and miR-182-5p) the first cycle of neoadjuvant chemotherapy were found to correlate with the therapeutic effects in breast cancer patients [147]. 

A study on miRNAs of EVs isolated from sera of patients with epithelial ovarian cancer (OC) reported that eight, including miR-92, miR-93, and miR-126, were highly expressed in patients compared to healthy individuals [148].

Recently, circRNAs have emerged as non-invasive biomarkers with high sensitivity and specificity for cancer [149]. These circRNAs are generated from linear pre-messenger RNAs after a back-splicing mechanism when 3′ and 5′ ends bind to form a continuous loop, covalently closed [127]. CircRNAs are resistant to RNAse activity since they lack 5′ and 3′ ends and have a longer half-life than canonical linear RNAs. Since circRNAs have a significant role in tumorigenesis and cancer development, they have been studied in cancer diagnosis and prognosis. Cancer patients show circRNA expression levels in the ratio of 2:1 compared to healthy controls [150]. Circulating circRNAs of the peripheral blood are mainly enriched in EVs [147]. CircRNAs function as miRNA sponges; they could sequester miRNAs by protecting target genes from repression by miRNAs. One circRNA can sponge different miRNAs, establishing intricate and precise regulatory networks [151].

Since EVs may be isolated from body fluids and their lipidic membranes protect circRNAs from RNase degradation, EV-circRNAs have potential as biomarkers for early diagnosis and prognosis. An increasing number of studies have reported the abnormal expression of circRNAs in different cancer types, for instance, in OC; it was demonstrated that CircRNA-051239 expression is increased in tissues and EVs isolated from the plasma of OC patients; circRNA acts as ceRNA by sponging miR-509-5p to induce Serine protease 3 (PRSS3) expression. Cdr1as is downregulated in serum EVs from cisplatin-resistant patients; the circular RNA Cdr1as (cerebellar degeneration-related protein 1 antisense) sensitizes OC to cisplatin by regulating the miR-1270/SCAI signaling pathway [152]. Moreover, aberrant expression of long non-coding RNA (lncRNA) contributes to tumorigenesis and metastasis, and lncRNAs are selectively sorted into cancer EVs. The expression of many lncRNAs is tissue-specific, so the identification of EV-lncRNAs may facilitate cancer diagnosis [153].

EVs have attracted research interest due to their role in carrying specific proteins and lipids for different cancers. To check EV proteins as biomarkers can be useful for early diagnosis of cancer. For instance, clinically, EV-PD-L1 (programmed death ligand 1) is considered a potential predictor of response for anti-PD-1 therapy in patients with melanoma and NSCLC [154]. Furthermore, in EVs isolated from patients with prostate cancer, higher levels of CD81 and prostate-specific antigen (PSA) were detected, which could discriminate prostate cancer patients from benign prostatic hyperplasia and healthy individuals [155]. In addition, the identification of CD24 and EGFR in OC-derived EVs has been proposed as an alternative approach for early detection [156].

The protein sorting and localization within EVs is often associated with regulated biological processes that reflect the parental cell’s identity, molecular state, and external-stimulus response. For instance, cytokines associated with EV surfaces are more stable than free soluble cytokines [157].

EV lipids may also be a promising source of biomarkers for cancer detection. The lipid composition of cancer EVs has mainly been studied in melanoma, colorectal, breast, and prostate cancer cell lines. Lipidomic analysis of urinary EVs from prostate cancer patients has suggested that a combination of two phosphatidylserines and a lactosylceramide can distinguish between healthy individuals and prostate cancer patients [158]. High levels of sphingolipids have also been noticed in breast cancer tissue when compared to normal breast tissue through lipidomic analysis using mass spectrometry [159]. A recent study reported that EVs released by blood plasma can be a source of lipid biomarkers for breast cancer detection. Among the lipids contained in these EVs, phosphatidylcholines (PCs) and phosphatidylethanolamines (PEs), which have also been detected in breast cancer tissues, were the highly abundant lipids [160]. Table 2 summarizes the potential EV biomarkers reported in this section.

Overall, this evidence indicates that EVs are potentially good biomarkers for cancer diagnosis, albeit some technical and functional pitfalls must be addressed to use EVs routinely at both pre-clinical and clinical levels.

**Figure 4:**
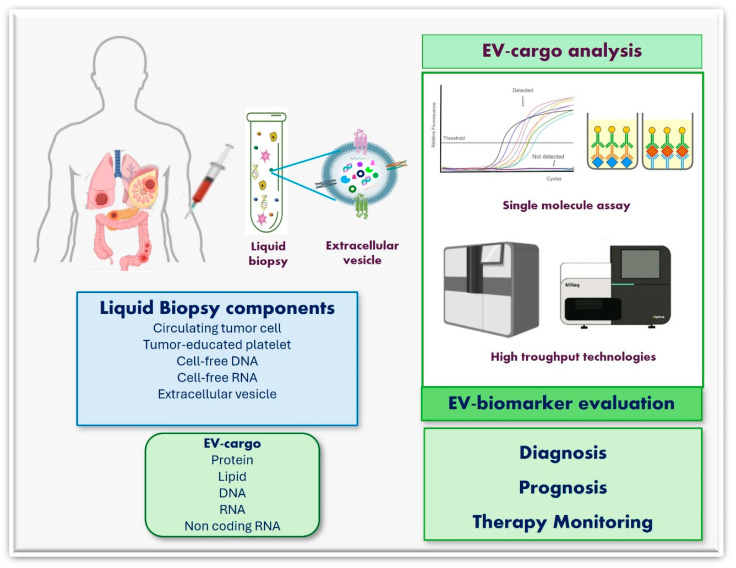
Liquid biopsy allows the use of EVs in clinical management of cancer.

Researchers are currently questioning if understanding EV surface composition is beneficial for clinical molecular biology. Although the improvement of high-throughput omics technologies has allowed in-depth characterization of EV contents, the spatial distribution of the bioactive molecules within EVs is not well known. On EV-surface, several molecules can be integral, or peripheral associated with the membrane. The corona that surrounds the EV membrane influences an interactive and dynamic surface area that helps EV interactions with the microenvironment. The EV corona is composed of surface molecules that reveal the identity of parent cells; this property is of great value in the diagnostic liquid biopsy scenario [161]. Therefore, in clinical molecular biology, EVs represent potential biomarkers. Designing an EV-based non-invasive liquid biopsy is essential for cancer diagnosis, prognosis, and monitoring (Figure 4).

Currently, different clinical trials to investigate EVs and their cargos as accurate tools for cancer diagnosis are ongoing. Several clinical studies are evaluating EVs obtained by liquid biopsy for early cancer diagnosis, especially in pancreatic (NCT05625529), rectal (NCT04852653), breast (NCT05417048), and lung (NCT05469022) cancer. Moreover, clinical trials to analyze EV-ncRNAs as biomarkers for cancer diagnosis are ongoing. For instance, serum exosomal lncRNAs are being evaluated as potential biomarkers in lung cancer diagnosis (NCT03830619); other clinical studies are aiming to validate an exosome-based miRNA signature for non-invasive and early detection of pancreatic ductal adenocarcinoma (NCT04636788) and to analyze the expression of miRNAs and lncRNAs by NGS in patients with high-grade serous ovarian cancer as biomarkers for the detection and prognosis of ovarian cancer (NCT03738319) (https://clinicaltrials.gov, accessed on 20 August 2024).

## 6. The Role of EVs in Cancer Diagnosis: EVs as New Therapeutic Options

EVs have several advantages over conventional synthetic shuttles, opening new frontiers in drug delivery. EVs are being studied for the delivery of therapeutic cargos to specific cells or tissues, exploiting their intrinsic tissue-homing capabilities. EVs can travel long distances and transfer their cargos to specific cytotypes through ligand–receptor binding. Another interesting feature of EVs is their capability to cross tissue barriers. EVs isolated from blood are promising, safe drug delivery carriers owing to their inability to trigger innate immune responses. In this section, we describe only the most innovative approaches that involve the use of EVs as shuttles of immunotherapeutic agents.

### 6.1. EV-Based Cancer Immunotherapy

EVs have good potential in cancer immunotherapy thanks to their crucial role in the interaction between cancer and immune cells [162].

The potential of EVs in immunotherapy was first reported in the 1990s, when it was demonstrated that dendritic cell (DC)-derived EVs induced specific cytotoxic activity of T lymphocytes in vitro and in vivo [163].

New strategies focus on the application of EVs to increase anti-cancer immune responses or to overcome immunosuppressive activities. EVs express antibodies against checkpoint proteins; thus, they can be considered potential cancer immunotherapies. Therefore, EVs can be shuttles of clinical monoclonal antibodies; furthermore, cancer immunotherapeutic agents, such as ICIs (immune checkpoint inhibitors), tumor antigens, tumor peptides, cytokines, and interleukins, can be loaded into EVs to improve their targeting efficacy against tumor tissues.

EV-based biomarkers have been used to monitor therapeutic response through LB in cohorts of patients treated with ICIs. These studies focused on miRNAs and proteins in EVs, especially in lung cancer and melanoma. In lung cancer, in pre-treatment plasma EVs from advanced NSCLC patients, high levels of miR-200c-3p, miR-21-5p, and miR-28-5p have been reported in non-responder patients to anti-PD1 or anti-PDL1 therapy. The combined evaluation of miR-199a-3p, miR-21-5p, and miR-28-5p predicts the response to immunotherapy better than PD-L1 immunohistochemistry [164]. In NSCLC, miR-320b, miR-320c, and miR-320d, at baseline, can predict progressive disease versus a partial response to ICIs. In patients with a partial response to ICIs, in post-treatment plasma EVs, miR-125b-5p, a T-cell suppressor, was decreased when compared to pre-treatment samples [165]. Recent studies have investigated EV protein biomarker dynamics in NSCLC and reported that an increase in PD-L1 in EV following treatment with ICIs is associated with poor response and survival outcomes [166].

According to this clinical finding, EVs containing PD-L1 released by lung cancer cells can reduce T-cell activity and promote tumor growth [167]. Other EV-associated proteins involved in neutrophil degranulation, such as annexin A2 and S100A8/9, increased with ICI treatment in responders, while in non-responder patients, they decreased [168].

In melanoma EVs, dynamic changes in PD-L1 at mRNA and protein levels have been evaluated as biomarkers related to ICI response [169]. In order to monitor immunotherapeutic response, PD-L1-mRNA expression in EVs isolated from plasma of melanoma and NSCLC patients was evaluated. Decreased *PD-L1* levels have been observed in patients with a partial or complete response; conversely, an increased expression of EV-*PD-L1* was observed among non-responders following treatment with ICIs [154]. PD-L1 protein levels in EVs were significantly higher among patients with metastatic melanoma and non-responders to ICIs. In contrast, increased levels of EV-PD-L1 during early treatment with immunotherapy were found, predicting increased response rates in melanoma patients [169]. In melanoma, next-generation sequencing (NGS) has been used to identify EV biomarkers related to immunotherapy response. RNA-seq profiling of plasma EVs isolated from metastatic melanoma patients showed a decrease in several transcripts and pathways related to CD28 costimulatory, T-cell receptor, and CTLA4 signaling during treatment with ICIs in non-responders. Many transcripts related to immunotherapy resistance (CD1A, MAP2K4, TRBV7–2, and IGFL1) were enriched in pre-treatment samples of non-responders [170]. However, a flow cytometry analysis of EVs collected from metastatic melanoma patients treated with an immunotherapeutic agent, the monoclonal antibody ipilimumab, showed, at baseline, that the levels of EV-PD1 and CD28 from T-cells were associated with improved PFS and overall survival [171].

Recently, it was reported that, under ICI treatment, an increase in myeloid-derived suppressor cells (MDSCs), especially monocytic MDSCs, predicted ICI resistance in metastatic melanoma [172].

The role of EV-PD-L1 as a biomarker for immunotherapy response showed different predictive associations with respect to soluble PD-L1 in multiple tumors [173]. Overall, these data indicate that plasma EVs derived from both tumor and immune cells allow a better stratification of patients’ responses to immunotherapy.

Currently, immunotherapy clinical trials integrating the assessment of LB biomarkers in lung cancer and melanoma, such as EV-PD-L1 and miRNAs in NSCLC (NCT04427475) and EV-PD-L1 in melanoma (NCT05744076) (https://clinicaltrials.gov, accessed on 20 June 2024), are ongoing [174].

### 6.2. Drug Delivery Research

EVs provide several advantages in drug delivery, as they transfer their cargos, have a long-range impact, and can bypass and permeabilize the blood–brain barrier. In comparison with other nanocarriers available for drug delivery, EVs are non-toxic, non-immunogenic, and can avoid phagocytosis and the process of engulfment by lysosomes [175,176]. EVs can be used to deliver drugs or other inorganic particles to specific targeted sites, such as the central nervous system, the pancreas, the liver, and eyes. There are a variety of methods for loading different therapeutic agents (physical, chemical, and biological) into EVs. These compounds can be also loaded into donor cells, as the composition of EVs is highly controlled within cells. Two major strategies for loading EVs with drugs can be performed: endogenous and exogenous [177].

1. ***Endogenous approach:*** Donor cells can be incubated with bioactive molecules of small molecular weight. Cargos, after passive diffusion across the plasma membrane, can concentrate in the cytoplasm; thus, the donor cell will utilize its natural mechanism to package drugs into vesicles, and, after appropriate stimulation, such as hypoxia or heat, EVs carrying these molecules are generated. Exogenous nucleic acids are usually loaded into donor cells using a gene transfection approach; the cells are transfected with DNA plasmid vectors, non-coding RNAs, etc., that are easily packaged within EVs by the natural processes of biomolecular synthesis. The approach is simple, but it can result in poor loading and is thus not suitable for wide application [178].
2. ***Exogenous approach:*** This is an alternative to encapsulation during vesicle biogenesis, as EVs previously isolated from donor cells are purified to obtain concentrated EVs. Then, vesicles are incubated with the drug of interest, which can easily penetrate inside and localize in the lumen. The loading strategy relies on a simple step of incubation (passive drug loading) or exposure of EVs to active stimuli (active drug loading), for example, electroporation, saponin permeabilization, freeze/thaw cycles, sonication, hypotonic dialysis, and/or extrusion. Currently, the principal method used for loading siRNA is EV electroporation. The application of high-voltage electricity to an EV suspension generates temporary pores in the membranes, through which therapeutic compounds can be internalized in EVs [177].

The choice of donor cells depends on the nature of the parental cells; nanocarrier safety in clinical applications is a primary concern [63,64,65,66,67]. Several studies suggested that not all EVs are ideal as nanoshuttles. Drug delivery capacity and efficiency depend on the size, yield, composition, and surface proteins that mirror the cell sources and tissue of origin [68]. The major types of EVs that are used come from dendritic cells, red blood cells, human milk, food products, prokaryotes (Gram-positive and Gram-negative bacteria, attenuated bacterial strains—with no substantial pathogenicity or toxicity and archaea) edible plants, and cancer cells. Tumor cells may be a double-edged weapon when used to shuttle therapeutic agents, as their EVs could carry the potential risk of exacerbating cancer progression or confer drug resistance [179,180].

EVs with specific loaded cargos release their contents into the recipient cells by different mechanisms, and they can induce cellular phenotypic switching. EVs are ideal nanocarriers for drug delivery and can be engineered. An approach to modify EVs and improve their specific targeting ability is membrane modification—chemical modification to the surface of EV membranes [181,182]. Surface-modified EVs for cerebral ischemia therapy have been generated [183]; paclitaxel-loaded EVs were modified with PEG and AA (ligands) to improve their circulation time in the blood and allow them to target pulmonary metastases [184]. To increase the delivery function of EVs, Sato et al. attempted to fuse the membranes of EVs with liposomes using the freeze/thaw method, generating hybrid EVs as novel biological nano-transporters (bio-nano-transporters) [185].

An important prospect for future therapeutic approaches is the generation of engineered vesicles to improve the efficacy of anti-cancer therapy. As an alternative to natural EVs, biomimetic and bioinspired nanovesicles and organic–inorganic hybridized and synthetic nano-formulations called artificial EVs have been constructed and employed. Synthetic nanovesicles, fabricated by forcing cells through microfluidic channels, have been assessed for augmented proliferation in murine skin fibroblasts [186]. EVs loaded with doxorubicin, a widely used chemotherapeutic agent that inhibits angiogenesis and controls tumor growth, can be easily internalized, avoiding the role of the efflux pump [187]. Engineered EVs were actively delivered to the ischemic area, and infarct volume, apoptosis, and BBB destruction were attenuated. EVs are designed RNA nanocarriers [188]. The current reports on EVs as drug delivery vehicles are mainly related to the delivery of small nucleic acids, such as miRNAs and siRNAs, or low-molecular-weight drugs. Lin et al. successfully encapsulated large nucleic acids, and CRISPR/Cas9 into hybrid EVs produced by incubating primitive EVs with liposomes, for the treatment of various diseases [189].

Nevertheless, there are a lot of difficulties and certain drawbacks in maintaining the immune status of carriers. For detailed overviews of these topics, the reader is referred to recent reviews [190,191].

**Figure 5:**
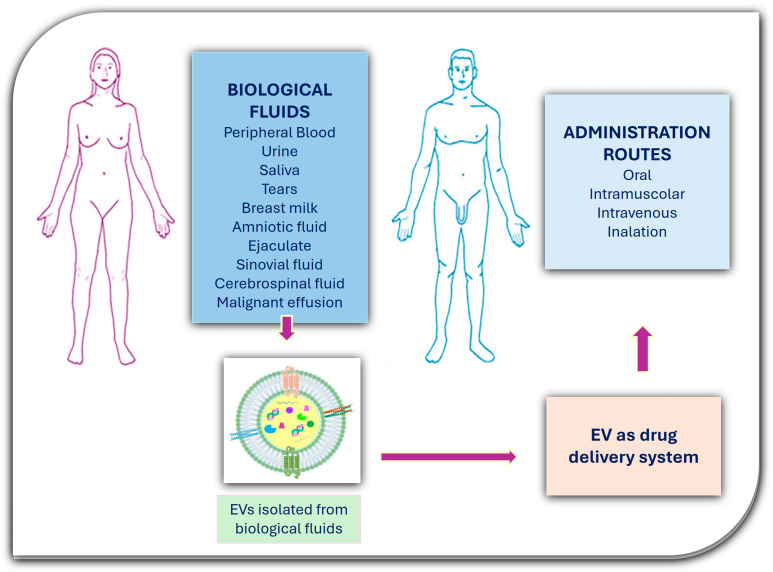
EVs collected from different biological fluids can be used as drug delivery systems via various administration routes.

One other important aspect to improve delivery of chemotherapeutic drugs is to ensure the accumulation and biodistribution of EVs. It was reported that EVs can quickly dissolve from the systemic circulation when injected intravenously in mice [192]. The route of administration is a crucial factor affecting the safety and efficacy of EVs. EVs can be administered via various routes, for example, intravenous, subcutaneous, intraperitoneal, nasal, and oral administration, among others. EV administration locally by intraperitoneal and subcutaneous injections results in a significantly lower EV accumulation in the liver and spleen. Intratumoral injection can deliver drug-loaded EVs at higher concentrations to target cells, increasing the potency of the treatment [193]. However, as intratumoral delivery requires access to the tumor site, this approach may be invasive for some organs and specific tumors and will be less desirable when repeated treatments are needed. The intranasal administration route has been shown to be more effective than other administration routes to deliver drugs to the central nervous system, particularly by circumventing the challenges of delivery across the blood–brain barrier (BBB). It is necessary to choose the correct way of administration, as different routes can affect the biological distribution of drugs in vivo. Among the different routes of administration, we report a novel strategy that consists in the nebulization of EVs as inhalable dry powders (Figure 5).

- *Inhalable dry powders of EVs*

Delivery of anti-cancer drugs directly into the lungs by inhalation is non-invasive and patient-friendly and can facilitate the arrival of therapy in lung cancer tissues, increasing the efficacy and reducing doses and frequency of administration and off-target effects. Lung delivery is commonly achieved using nebulizers and dry powder inhalers (DPIs) [194]. Nebulizers are the devices of choice for the aerosolization of therapeutics in solution or suspension to avoid additional formulation development (e.g., drying) and enable fast advancement to clinical trials [195]. Since nebulization may cause damage to lipid-based nanocarriers and leakage of molecules encapsulated as cargo, dry powder formulations are more stable compared to their liquid counterparts [196]. Nebulization technologies have evolved with time and are routinely used. Through a nebulizer, a drug can be deposited directly into the respiratory tract, making this route an attractive delivery option. Regarding aerosol devices, the vibrating mesh nebulizer (VMN) shows higher efficiency than other nebulizers, due to the low residual drug volume and improved drug stability.

A few studies indicate that EVs loaded with genetic materials could be delivered into the lungs via aerosol, transferring functional ncRNAs to the lungs and targeting macrophages and airway epithelial cells [197].

The potential of delivering mRNA encoding SARS-CoV-2 spike protein loaded in lung-derived EVs to the lungs has been investigated [198]. Moreover, siRNA-loaded EVs were delivered into murine airways via nebulization [197].

Recently, it was also demonstrated that inhalable dry powders of miRNA-laden EVs can be used for pulmonary delivery as a therapeutic option in primary lung cancer and in lung cancer metastasis of breast cancer [199]. These reports indicate that EVs with exogenous ncRNA delivered into the lungs can represent a potential treatment of primary or metastatic lung cancers, preserving the integrity and functions of EV-ncRNAs.

- *Three-Dimensional bioprinting of EVs*

Recently, three-dimensional bioprinting (3DBP) has been developed as a promising strategy for engineering complex tissue scaffolds for biomedical applications. A few studies have reported the possibility of generating EVs via 3D bioprinting (3DBP). To date, two reviews and ten published studies utilizing 3D bioprinted sEVs for tissue engineering have highlighted the potential of “cell-free” regenerative therapy by combining bioprinting and EVs [200].

The 3DBP strategy of EVs is described in tissue engineering and regenerative medicine applications. EVs can be engineered to shuttle exogenous cargo such as nucleic acids and proteins of therapeutic importance. The 3DBP of EVs offers great potential in the engineering of implantable constructs for the localized transport of EV-based therapeutics with precise spatiotemporal control.

Important challenges, such as selection of an appropriate bioink, pattern resolution, engineering-defined EV gradients, spatial presentation, EV-release kinetic modulation, and EV stability and storage conditions, must be addressed for successful translational medicine. Currently, 3DBP-EVs are studied in a regenerative medicine approach: 3DBP-EVs tissue engineering. EVs derived from endothelial cells can be used as bioink additives and more effectively delivered in vivo when combined with a 3D bioprinting strategy, supporting the rapid neovascularization of bioprinted, cell-free grafts [201].

Although bioprinted EVs hold great potential, several challenges need to be taken into account. (a) An important aspect to investigate is whether bioprinted EVs can perform a therapeutic role at specific sites with an appropriate release profile. Crucial aspects of designing bioinks are constant EV release and their mechanical properties. The selected bioink and bioprinted structures should be degraded after EV release to specific districts for host cell recruitment. (b) Functionalizing EVs and their binding to the printed scaffolds is crucial to obtain targeted cellular responses after EV release from bioprinted scaffolds [202].

Among less than ten studies, five studies employed bioprinted EVs in pre-clinical animal models [203,204]. Further studies are needed to support the in vivo functional role of bioprinted EVs.

## 7. Considerations and Challenges in EV Research

EVs are natural lipid membrane particles constitutively released by all living cells, including plants, animals, and microorganisms. EV cargos are complex and well-coordinated mixtures of biomolecules. They are involved in many biological activities and are essential mediators of intercellular communication, carrying bioactive molecules across cells and biological barriers under both healthy and pathological conditions. EVs exerting a plethora of biological functions have aroused wide interest in biomedicine, nanomedicine, and medical sciences for diagnosis, targeted therapy, and drug delivery.

EVs are considered good candidates to deliver therapeutic compounds thanks to their low immunogenicity, good biocompatibility, and biological regulatory function. Thus, drug delivery is one of EVs’ most promising applications in cancer therapy and other pathologies. As aforementioned, EVs can deliver their cargo, protecting it from degradation. Therapeutic EV research has seen a great expansion in the past decade, from in vitro studies to pre-clinical models to clinical trials registered in the clinical trial database of the National Institute of Health accessed on 20 June 2024 (www.clinicaltrials.gov or https://clinicaldata.ema.europa.eu). EVs may be useful for the early and non-invasive detection of cancer to identify cancer patients and monitor how they react to therapy [205]; some clinical applications have now been approved by the United States Food and Drug Administration (FDA).

Tumors and other types of diseases produce more EVs than healthy cells; thus, there is a link between the amount of EVs and the presence of disease [206]. Also, carrying a cargo that mirrors genetic or signaling alterations of parental cells, EVs provide useful biomarkers for a variety of human diseases as non-invasive diagnostic tools. There is differential expression of EV-RNA and proteins derived from normal and diseased cells [207]. To date, circulating miRNAs have mainly been used to carry out effective diagnosis and prognosis of numerous diseases [208]. EVs are sources of tumor biomarkers, such as proteins, lipids, and several nucleic acids, which provide information about donor cells, cancer stage and progression, aggressiveness, and the microenvironment [53,149,153].

EVs can be valuable as diagnostic, predictive, and pre-symptomatic disease-state biomarkers. Clinical research and applications in clinical tests focus on the accuracy of isolation procedures, stability, obtaining high-quality EVs separated in their native state, and the convenience of methods.

Since the first use of UC, purification methods have evolved rapidly for the optimization of EV isolation, and recent advances in EV characterization have facilitated the expansion of knowledge about them and the definition of disease-specific cargos carried by these messengers. However, before using EVs in potential therapeutic applications, various challenges need to be overcome.

- The top priority is to use robust purification methods, as the extracellular environment in biofluids, such as plasma, urine, and saliva, used as non-invasive sources, is rich in proteins, lipoproteins, aggregates, cell debris, and other contaminants. It is necessary to consider the characteristic of the sample to be analyzed because each body fluid has its own composition and biophysical properties. The choice of isolation method for EVs can have an impact on the quantity as well as the cargo composition of the obtained EVs [209].
- Moreover, some of these biomaterials contain EVs from all around the body, and it is extremely difficult to determine the origin of vesicles isolated from these biomaterials. Microvesicles in plasma are mainly from platelets, but other sources of EVs in blood plasma are endothelial cells, monocytes, lymphocytes, and erythrocytes [210], and less than 1% of EVs originate from tissues such as smooth muscle cells. EVs’ size heterogeneity and the diversity of their surface proteins, due to the wide range of producing cells, severely limits the purity of extracted EVs and their promising use.
- Due to the biogenesis of these vesicles, there are still methodological difficulties in performing experiments to identify the tissue specificity of isolated EVs, nor it is possible to differentiate some membrane and cargo markers which are specific to EVs or which can be identified both in cells and in EVs from the parental cells.
- The minimal relative abundance of EVs in body fluids makes their isolation and analysis more difficult.
- Preservation of the structural integrity of EVs is necessary to allow proper characterization of them. EVs are sensitive to storage conditions, so the stability and integrity of biomarkers need to be preserved. EVs isolated from diverse sources may require different storage conditions. New appropriate storage conditions such as lyophilization methodologies and handling methods to maintain the stability and integrity of EVs need to be developed [211,212,213].

Currently, a single technology is not able to cover the full range of analyses for EVs. In recent years, appropriate combinations of two or more methods to extract and purify EVs have been developed to improve these limitations [214]. SEC, preserving the integrity and natural biological activity of isolated EVs, offers several advantages in comparison to UC; hence, the combination of both methods facilitates the preparation of EVs for clinical use. Recent studies for EV isolation used SEC and UC, employing cell lines; this preparation may be a limitation for potential clinical translation, as cell lines are not always a good mirror for a real clinical scenario. For example, physical characterization methods are often unable to differentiate EVs from different sources; thus, the use of immune-capture detection provides evidence of antigens or genomic study of different EVs [215]. The characterization method is selected according to the scope of the analysis. TEM is the standard method to follow to identify the morphology and confirm the sample purity. If the purpose is to analyze the size and morphology of particles, NTA is sufficient. Western blotting and ELISA can be used to detect and identify EV proteins with respect to their role. However, selection of cell-type-specific markers based exclusively on transcript or genomic enrichment in a particular cell type is often not sufficient. Biochemical characterization may be performed with proteomic and lipidomic approaches, but these studies are often limited by EV heterogeneity. NTA and DLS are also limited, as they lack the sensitivity needed to validate the observed EV heterogeneity [45]. Moreover, some of these procedures are time-consuming, requiring professional skills as well as laboratory facilities and benchtop instrumentation. Many attempts have been made to achieve good results exploring recent technologies, including acoustic techniques, microfluidic-based platforms, membrane-based platforms, and single-molecule localization, to efficiently separate and characterize EVs. Microfluidic chips show high modification efficiency, high integration, and controllability [216]. Electrochemical nano-sensors have been shown to be quite useful due to their low maintenance requirements, excellent precision, and consistent reproducibility [80,217,218]. Plasmonic-based biosensors can detect EVs in real time and in a nondestructive manner, which is important for preserving the integrity of EVs. Nevertheless, plasmonic-based biosensors have many drawbacks, including the requirement of costly equipment and the possibility of interference from other biomolecules. There are still technical and economic difficulties, and these novel strategies need to be perfected and optimized. Each EV isolation/characterization method displays its own advantages and disadvantages; and each method seems often not completely appropriate for the specific scientific purposes and/or clinical studies.

EVs can be used as drug delivery agents: characteristics such as the precise therapeutic requirements, i.e., the chemical nature of the drug, the mode of loading, the targeted disease site, and the mechanism of action, have important implications for drug loading efficiency. Recently, post-insertion of siRNA into EVs by conventional bulk electroporation has been successfully developed with a good performance superior to that of synthetic nanocarriers in several mouse models for central neuron and cancer treatment; encapsulating certain drug molecules such as miRNA and siRNA into EVs can specifically kill tumor cells and avoid damage to normal cells. However, an open question is whether loading exogenous cargo may also interact with endogenous cargo, creating problems associated with off-target effects. Additional studies are needed to understand if the different methods of modification and functionalization of EVs designed by researchers could compromise the biological functionality. At present, engineered EVs, such as biomimetic and bioinspired nanovesicles, are in development. EVs can be easily integrated with organic nanoparticles, such as liposomes for membrane fusion; also, diversified hybrid inorganic nanoparticles with EVs for both therapy and diagnosis have been designed. However, the biological application of nanomaterials still needs more research to optimize and improve their performance in vivo, enhancing the targeting and preserving the bioactivity and biodegradability of EVs; attention to their safety and biocompatibility also needs to be evaluated to ensure that the inorganic/organic materials do not accumulate in the body. Moreover, the preparation technology introduces specific difficulties to industrial production: large numbers of cell cultures (such as mesenchymal stem cells and dendritic cells that are known to secrete relatively high numbers of EVs) and days of incubation followed by purification and nucleic acid loading are required to generate gene-containing EVs in sufficient quantities for repeated use in human therapy. We direct the reader to the following recent reviews and position papers [219,220,221,222].

If a biomarker should be used in clinical applications, there should be no statistically significant differences between different detection institutions, detection methods, and researchers. Thus, to improve the consistency and repeatability of results, a criterion should be established for using different specimens and collection tubes, even for the same blood matrix [223].

## 8. Conclusions and Outlooks

EVs have received wide attention from scientific communities, since they show great promise for new treatments of various diseases, particularly cancer, given its strong impact on human society. EVs can be developed as new therapeutic alternatives to overcome the limits of conventional radiotherapies and chemotherapies. EVs can be diagnostic and prognostic indicators, as their contents reflect their producer cells.

Reviewing the latest research progress, this paper summarizes the main features of EVs; it provides a comprehensive overview of the isolation, characterization, and recent standardization strategies used to select and collect EVs from cells, focusing on their advantages, limitations, and potential areas of application. Selected aspects of their main applications in cancer studies are given.

Further efforts are needed to realize the development of an ideal technique, a universal and efficient isolation method, achieving upscaled production that can be easily used in future clinical applications; many knowledge gaps also need to be filled to validate EVs’ utility for their routine use in clinical practice. New approaches, such as super-resolution microscopy, that allow direct visualization of single EVs, analysis of their surface proteins, and in vivo tracking and real-time imaging, are expected to improve the accurate detection of specific EV subpopulations originating from different cell types or from a single cell source and the exploration of the spatiotemporal heterogeneity of EVs. These specialized analysis methods have increasingly employed fluorescent affinity tags; however, they are still in their infancy. Animal studies to elucidate the biodistribution of administrated EVs and the exploration of the spatiotemporal heterogeneity of EVs, their storage parameters, biocompatibility, routes of administration, therapeutic efficacy, and safety through in vivo studies need to be undertaken urgently. With the goal of improving therapeutic pharmacokinetic properties and effects, several strategies to engineer EVs as intelligent, reproducible drug delivery systems are being developed. Further insights are needed to completely understand these strategies and increase loading efficiency, in the expectation that EV use will become routine in the future.

This is a rapidly growing area of research; thus, it is expected that, in the future, automated platforms will be developed for the simultaneous separation and detection of native EVs. The combination of innovative methodologies, such as synthetic biology, nanotechnology, machine learning, and artificial intelligence, may also improve the design of simple, economical, and efficient chip laboratory equipment and standardized analytical systems with the aim of diagnosing cancer from liquid biopsies, taking advantage of their use in personalized medicines.

High technical rigor is required; authorized guidelines for the clinical routine use of EV biomarkers need to be published, considering also ethical and clinical implications. Different fields of sciences are involved, and thanks to interdisciplinary approaches and the collaboration between researchers, clinicians, ethicists, and policymakers, this area will advance, providing novelty and optimization of traditional therapeutic and diagnostic practices, contributing to innovative solutions for personalized cancer medicine and various health problems.

## Figures and Tables

**Table 1:** A summary table describing various commonly used EV characterization methods along ith their advantages and limitations.

Methods	Principle	Purpose	Advantages	Disadvantages	Ref
**Dynamic light scattering** **(DLS)**	EVs are challenged with laser beam, and scattered beam of light is captured at certain variable angle by detector. The size of the particles is determined by measuring the random changes (resulting from relative Brownian movements of particles) in the intensity of scattered light from sample suspension. Intensity of scattered light in function of time	Size distribution in the range of 1 to 6000 nm, for EVs concentrations from 106 to 109 particles/mL. After analysis sample recovery is possible.	Useful for qualitative anaysis No preparation steps	Not efficient with polydisperse samples Not suitable for measuring complex EVs samples with large size range. Non specificity Difficulty to distinguish contamination from EVs	[123]
**Nanoparticle tracking anaysis** **(NTA)**	EVs are challenged with a beam of laser and forward scattered light which is captured (real time) by a microscope to calculate the sizes of particles based on their Brownian motion	Size detection in the range of 50 to 1000 nm, Measurement of distribution and concentration, NTA is compatible fluorescence detectors	Small amount of sample is required; possibility to get precise size distributions and their associated concentrations in 1-nm intervals. Detection speed is fast, and observation can be carried out in real time. analysis of EVs markers by fluorescent labeling.	Difficulty to distinguish contamination from EVs. Inaccurate if samples are aggregated and/or have different size distributions	[124]
**Scanning and Transmission** **electron microscopy** **(TEM-SEM)**	Imaging is performed using negative staining of EVs with heavy metals molecules. Immunogold labeling is used for staining specific structures. Cryo-TEM can be used for high resolution imaging. Scattered electron beam.	Direct visualization of EVs morphology, size measurement, and staining of specific structures	Requires a small sample amount. Semi-quantitative, can provide information of EVs distribution	Complicated operation and high requirements on sample preparation Sample preparation may lead to shape modifications	[122]
**Atomic force Microscopy** **(AFM)**	Uses a cantilever with a free end that touches the sample surface. The interaction forces between the probing tip and the EVs surface allow to obtain topographical information	3-D topography and diameter of EVs. Resolution at nanometric level can be achieved. useful for analysis of both dry and liquid samples; can be integrated with microfluidic devices	Useful for size distribution profile determination. No fixation or staining steps. Requires a small sample amount	Does not provide direct imaging of the EVs samples. Sample dehydration may lead to topography modifications	[125]
**Enzyme-linked Immunosorbent assay/Western blot** **(ELISA)/(WB)**	Based on the reaction between an antigen and an antibody	Detection of the marker proteins expression. WB involves lysis of vesicles and denaturation of proteins, so that both surface and internal proteins can be detected	Can qualitatively and quantitatively analyze marker proteins. Low sample volume, wide accessibility, ease of use	Time-consuming The Detection of marker proteins varies depending on the type of parental cells; not suitable for the detection of EVs markers in biological fluid	[126]
**Flow cytometry** **(FC)**	Vesicles are swept along by a liquid stream to align the particles in single line at the center of the stream where they are excited by a beam of laser. The scattered light is collected by detectors situated 180° (size data) and 90° (morphology) to the laser beam. Fluorescent light	Analysis of EVs with a lower size limit of 250 to 500 nm; ability.to distinguish vesicles that differ 200 nm in size. New technological progress has reduced the limit of detection to ~100 nm and the discrimination power to 100 to 200 nm. It can be coupled with fluorescent latex beads for surface marker	Qualitative and quantitative. Analysis speed fast; simple, required low concentration of the sample	Time-consuming Particle size cannot be measured	[127]

**Table 2:** A summary table describing new EV biomarkers as potential diagnostic tools.

Cancer Type	EV-Source	Bioactive Molecules	Ref
		miRNAs	
**NSCLC**	plasma	let-7, miR-223, miR-383, miR-192, miR-30e-3p, miR-301, let-7f, miR-572, miR-20b, miR-345, miR-30b, miR-30c, miR-103, miR-195, miR-221, miR-222	[142,143,144]
**Breast cancer**	plasma	miR-1246, miR-21, miR-9, miR-16, mi-429, miR-338-3p, miR-340-5p, miR-124-3p, miR-29b-3p, miR-20b-5p, miR-17-5p, miR-130a-3p, miR-18a-5p, miR-195-5p, miR-486-5p, and miR-93-5p	[145,146]
**Triple-Negative Breast Cancer**	plasma	miR-338-3p, miR-340-5p, miR-124-3p, miR-29b-3p, miR-20b-5p, miR-17-5p, miR-130a-3p, miR-18a-5p, miR-195-5p, miR-486-5p, and miR-93-5p	[147]
**Ovarian cancer**	serum, plasma	miR-92, miR-93, miR-126, miR-509-5p, miR-1270	[148]
		Circular-RNAs	
**Ovarian cancer**	serum, plasma	CircRNA-051239, Cdr1as	[152]
		Proteins	
**Melanoma, NSCLC**	plasma	PD-L1	[154]
**Prostate**	urine	CD81, PSA	[155]
**Ovarian cancer**	tissue	CD24, EGFR	[156]
**Lipids**			
**Prostate**	urine	phosphatidylserine, galactosyl ceramide	[158]
**Breast**	tissue, plasma	sphingolipid, phosphatidylcholine, phosphatidylethanolamine	[159]

## Data Availability

Not applicable.
